# Variation in Mesoderm Specification across Drosophilids Is Compensated by Different Rates of Myoblast Fusion during Body Wall Musculature Development

**DOI:** 10.1371/journal.pone.0028970

**Published:** 2011-12-14

**Authors:** Mirela Belu, Claudia M. Mizutani

**Affiliations:** 1 Department of Biology, Case Western Reserve University, Cleveland, Ohio, United States of America; 2 Department of Genetics, Case Western Reserve University, Cleveland, Ohio, United States of America; University of Otago, New Zealand

## Abstract

**Background:**

It has been shown that species separated by relatively short evolutionary distances may have extreme variations in egg size and shape. Those variations are expected to modify the polarized morphogenetic gradients that pattern the dorso-ventral axis of embryos. Currently, little is known about the effects of scaling over the embryonic architecture of organisms. We began examining this problem by asking if changes in embryo size in closely related species of Drosophila modify all three dorso-ventral germ layers or only particular layers, and whether or not tissue patterning would be affected at later stages.

**Principal Findings:**

Here we report that changes in scale affect predominantly the mesodermal layer at early stages, while the neuroectoderm remains constant across the species studied. Next, we examined the fate of somatic myoblast precursor cells that derive from the mesoderm to test whether the assembly of the larval body wall musculature would be affected by the variation in mesoderm specification. Our results show that in all four species analyzed, the stereotyped organization of the body wall musculature is not disrupted and remains the same as in *D. melanogaster*. Instead, the excess or shortage of myoblast precursors is compensated by the formation of individual muscle fibers containing more or less fused myoblasts.

**Conclusions:**

Our data suggest that changes in embryonic scaling often lead to expansions or retractions of the mesodermal domain across *Drosophila* species. At later stages, two compensatory cellular mechanisms assure the formation of a highly stereotyped larval somatic musculature: an invariable selection of 30 muscle founder cells per hemisegment, which seed the formation of a complete array of muscle fibers, and a variable rate in myoblast fusion that modifies the number of myoblasts that fuse to individual muscle fibers.

## Introduction

Sharp variations in embryonic size may account for the appearance of novel body patterns during evolution. Within the *Drosophila* genus, a number of related species that diverged recently have been previously reported to display large variations in egg size, and serve as excellent models to test how scaling affects the formation of morphogenetic gradients and cell fate specification [1], [2], [3]. One particularly attractive system to study the problem of scaling is the embryonic dorso-ventral (D/V) patterning. Among the advantages of this system is the fact that the readout of two opposing gradients (Dorsal/NFkB and Decapentaplegic/BMP4) can be visualized by well defined gene expression domains which establish the three primary germ layers, the mesoderm, neuroectoderm and ectoderm, in addition to several cell types within those domains[4], [5], [6], [7], [8], [9], [10]. Thus, one can precisely compare variations in the width of gene expression domains in small and large embryos and measure the relative domains of germ layers among different *Drosophila* species. Additionally, this system is particularly amenable to follow cell fates that develop into highly stereotyped tissues at late embryonic and larval stages, such as the nervous system and the somatic body wall musculature, derived from the neuroectoderm and mesoderm, respectively (reviewed by [11], [12], [13], [14], [15]).

If cells are allocated to particular germ layers as a function of how far these gradients can reach, then we expect that a variable spacing between the sources of D/V morphogenetic gradients should modify the number of cells allocated to each germ layer. However, a large body of evidence from the literature across divergent insect species suggests that the nervous system is not affected by embryo size. For instance, comparative anatomy of the ventral nerve cord between the fruit fly and other divergent insect species, including grasshopper and silverfish, revealed that they share a remarkably conserved organization with similar numbers and types of neural precursor cells, or neuroblasts, as well as identified neurons and connectivity patterns [16], [17], [18]. Therefore, while we should expect scale to affect patterning, there is a paradox in which organisms of diverse sizes can always allocate the same number of cells to the central nervous system, despite increases or decreases in total embryonic size. One possibility to achieve such extremely stable neuroectodermal domain would be if specification of other D/V germ layers were altered in order to account for the variation in embryo size. However, the possibility that changes in embryonic size could modify particular germ layers and tissues derived from these germ layers has not been tested yet.

Here we show that scaling changes the number of cells allocated within the D/V germ layers. We show that variations in embryo size among related *Drosophilids* impacts most notably the number of cells committed to become mesodermal precursors. In four species analyzed that vary in egg size in relation to *D. melanogaster*, we observe both expansions and retractions of the mesoderm. Thus, at least in these *Drosophila* species, the mesoderm specification can be highly variable while the mesoderm remains stable.

We next focused our analysis on the effects of such variations in the mesodermal domain at later developmental stages by following the fate of somatic mesodermal precursor cells that form the highly stereotyped larval body wall musculature [19]. The enlargement or shrinkage of the embryonic mesoderm across species could either lead to the development of novel fibers or elimination of specific muscle fibers per hemisegment. Alternatively, each individual syncytial muscle fiber could become smaller or larger by fusing with less or more myoblasts. Either outcome would allow us to determine whether there are mechanisms capable of compensating the observed variations in mesodermal size.

## Results

### Changes in embryo size across *Drosophila* species affect the width of mesodermal domain in blastoderm stage

**Figure 1:**
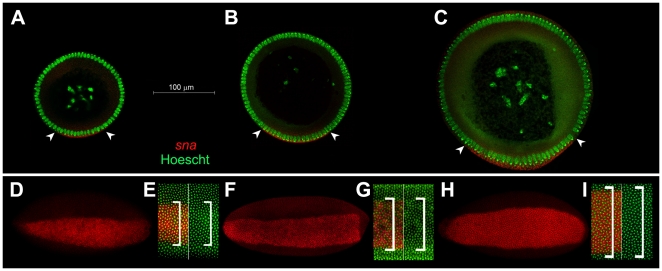
Mesodermal domain varies with embryo size. **A–C)** Cross-section of blastoderm stage embryos stained for mesodermal marker *snail* (*sna,* red) and nuclear dye Hoescht (green). **A**) *D. busckii*; **B**) *D. melanogaster*, and **C**) *D. sechellia.* Width of presumptive mesoderm is indicated by arrowheads and contains about 14 nuclei expression *snail* for *D. busckii* (**A**), 19 nuclei for *D. melanogaster* (**B**) and 26 for *D. sechellia* (**C**). Scale bar: 100 µm. **D–I)** Ventral view of whole mounted embryos adjusted to same size. *sna* is labeled in red, nuclei are labeled in green. **D)** *D. busckii* whole embryo and (**E**) high magnification detail of cells labeled with *sna* (left, *sna* and stained nuclei; right, nuclei only). **F, G)** *D. melanogaster*; **H, I)** *D. sechellia.* Brackets indicate extension of *sna+* nuclei.

To address the effect of scaling over the formation of the primary D/V germ layers, we selected four related *Drosophila* species previously described to have either increased or decreased egg size in comparison to *D. melanogaster.* Those species include *D. busckii*, which has the smallest egg of all species analyzed in this study and is the most divergent species [1]. We also selected *D. pseudoobscura* and *D. sechellia*, which have the smallest and largest eggs, respectively, out of other twelve *Drosophila* species previously analyzed [3]. *D. pseudoobscura* has an estimated divergence time of 46 mya from *D. melanogaster* [20], while *D. sechellia* is a sibling species of *D. melanogaster* that diverged very recently. Finally, we also analyzed *D. simulans,* another member of the Melanogaster subgroup of sibling species. *D. simulans* have eggs of slightly larger width than *D. melanogaster* (Chadha and Mizutani, unpublished data), but a significantly shorter length [3] and thus a modified overall geometry. Those three sibling species are especially attractive models of study given their very short divergence time of an estimated 5 mya between the ancestor of *D. melanogaster* and *D. simulans*, and only 0.3–0.5 mya between *D. simulans* and the newest species *D. sechellia* [21]. The difference in size between the smallest (*D. busckii*) and largest species (*D. sechellia*) in comparison to *D. melanogaster* can be seen in cross-section slices made in the trunk region of their embryos (Fig. 1A–C).

To visualize the embryonic mesoderm and neuroectoderm in different species, we used the mesodermal marker *snail* (*sna*) and neuroectodermal marker *short gastrulation* (*sog*) in *in situ* hybridization stainings [22], [23], [24]. The nuclei were counterstained with Hoescht and the ectodermal domain was identified by the absence of either *sna* or *sog* staining. In *D. melanogaster*, a total of 18 cells are allocated to mesoderm [22], [23], [24] (Fig. 1B, F, G). In contrast, the width of the mesodermal domain is decreased to about 14 cells in *D. busckii* (Fig. 1A, D, E). The mesodermal domain of *D. pseudoobscura* is also reduced to about 16 mesodermal cells (Fig. S1). In both *D. sechellia* and *D. simulans* the mesoderm is expanded to a total of 24–26 *sna+* cells (Fig. 1C, H, I; Fig. S1).

The variation of the mesoderm across the species analyzed contrasts with their invariable neuroectoderm, which have about 19 nuclei (Fig. S1). This invariability is expected since previous studies have shown that the number of neuroblasts in a broad range of divergent insects is conserved [16], [17], [18], and the maintenance of a neuroectodermal domain of precise width is a pre-requisite to establish correct numbers and identities of neural lineages along the D/V axis [25], [26], [27], [28], [29], [30]. Finally, our data is also in agreement with recent comparative analyses of expression patterns of *sog* in diverse *Drosophilids* at early blastoderm stage [31].

The results above indicate an interesting and unexpected property of D/V scaling, whereby the width of mesodermal domain is highly variable among related Drosophilids, while the neuroectodermal domain remains unchanged. At least in four species analyzed here, *D. busckii, D. pseudoobscura, D. melanogaster* and *D. sechellia*, the mesodermal domain expands according to an increase in embryonic DV axis. The comparison between *D. melanogaster* and *D. simulans* would constitute an exception to this trend, since *D. simulans* eggs have only a slightly larger DV axis measurement compared to *D. melanogaster*, but a much enlarged mesodermal domain (Fig. S1; Chadha and Mizutani, unpublished results). Nonetheless, we note that *D. simulans* eggs have a distinct shape and proportion from *D. melanogaster* eggs, as they are shorter in length [3], which may also be responsible for their different distribution of the Dorsal gradient in this species (Chadha and Mizutani, unpublished results). Thus, in general, changes in overall embryo size, as well as geometry, appear to have affected the final size of the mesoderm within a short evolutionary time.

### Expansion in mesodermal domain width correlates with increased numbers of somatic myoblast precursor cells

**Figure 2:**
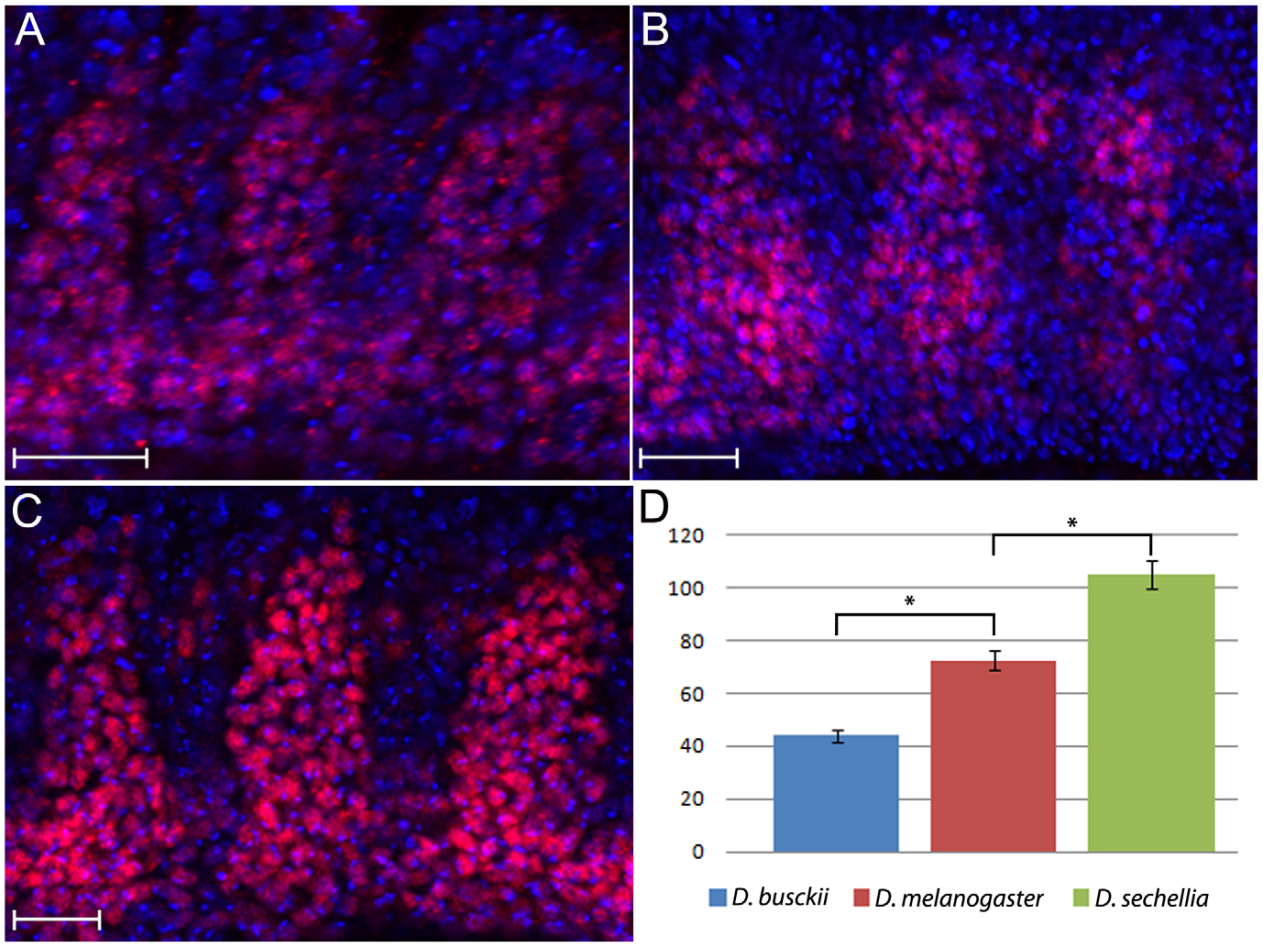
Expression pattern of Dmef 2 reveals differences in somatic myoblast numbers between Drosophila species. Lateral view of whole mounted mid-E12 stage embryos stained for anti-DMef2 antibody (red) and Hoescht (blue). External most layer of D-Mef2 positive cells are shown for (**A**) *D. busckii*, (**B**) *D. melanogaster* and (**C**) *D. sechellia*. **D**) The numbers of D-Mef2 positive cells are decreased to an average of 44 in *D. busckii* and increased in *D. sechellia* to an average of 105, in comparison to *D. melanogaster*, which has an average of 72 cells. Anterior to the left, dorsal is up. Scale bar: 20 µm. Sample size, n = 5 hemisegments. Asterisks indicate p-values of p = 0.0057 and p = 0.0059.

After specification, the mesodermal domain is further subdivided to originate a series of tissue types, including the somatic, visceral and cardiac muscles, as well as the fat body and blood [32], [33], [34], [35], [36]. To test whether a decrease or increase in mesodermal domains showed above would generate varying numbers of somatic myoblasts at later stages, we used an antibody against the transcription factor D-Mef2, which labels somatic myoblast precursors at mid-stage E12 [37], [38], [39]. We analyzed the species with greatest range in embryo and mesoderm size, *D. busckii* and *D. sechellia* and counted the total numbers of D-Mef2 positive cells present within the most external layer of hemisegments A2-A4. In *D. busckii*, there is an overall reduction of somatic myoblasts expressing D-Mef2 in comparison to *D. melanogaster* from an average of 72 cells to about 44 cells (Fig. 2A,B, D). In contrast, the number of D-Mef2 positive cells in *D. sechellia* has increased to an average of 105 cells (Fig. 2B,C, D). In conclusion, the increase in early mesodermal domain observed within the *Drosophila* species also leads to a corresponding increase in the numbers of myoblast precursor cells that will later constitute the somatic muscle fibers.

### Evolutionary conservation of the somatic body wall musculature

**Figure 3:**
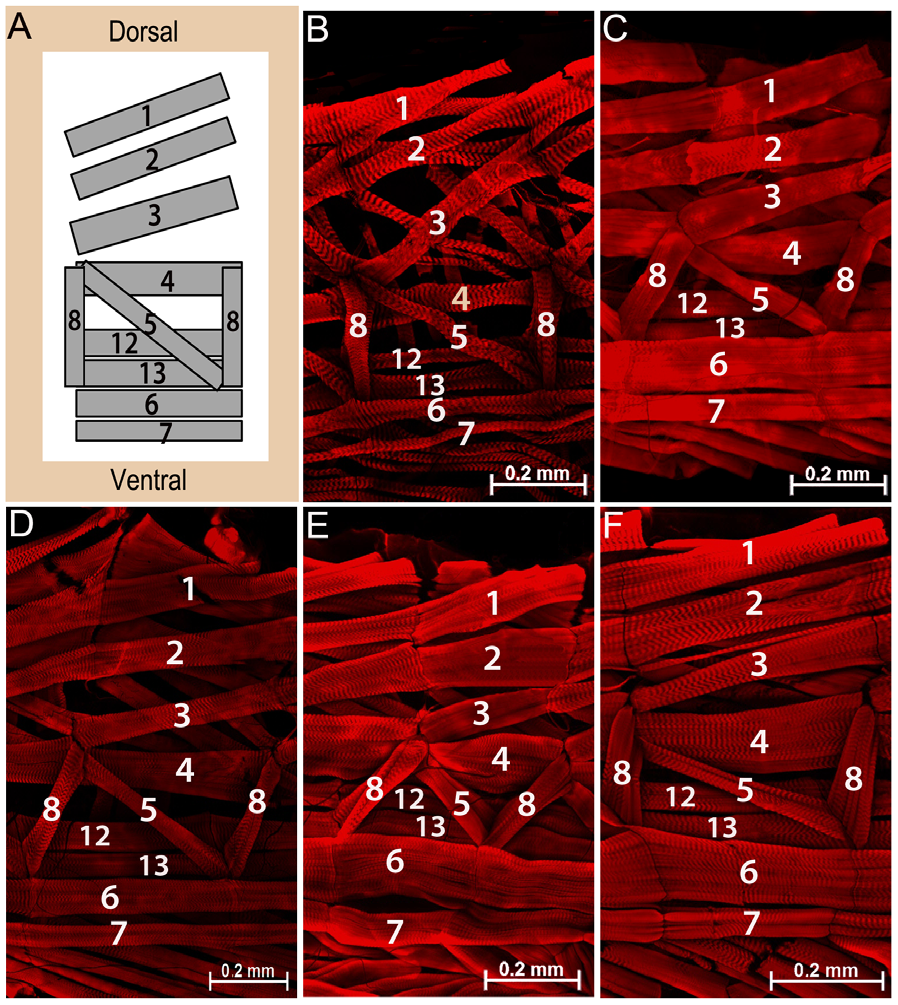
The pattern of internal muscle layer is identical in all species analyzed. **A**) Schematic representation of the larval body wall depicting the internal muscle layer of one abdominal hemisegment, as previously described for *D. melanogaster* (adapted from Bate, M., 1990 and 1993). Ventral and dorsal positions indicated in (**A**) also correspond to orientation of images shown in (**B**–**F**). Anterior is to the left. Dissected L3 larva of **B**) *D. busckii;* **C**) *D. pseudoobscura;* **D**) *D. melanogaster;* **E**) *D. simulans* and (**F**) *D. sechellia* species. The muscle fibers were stained with phalloidin (red). Each internal muscle fiber (indicated by its corresponding number) is present in all species, and displays similar orientation and attachment site as the stereotyped pattern described for *D. melanogaster* (**A, D**). Scale bars: 0.2 mm.

**Figure 4:**
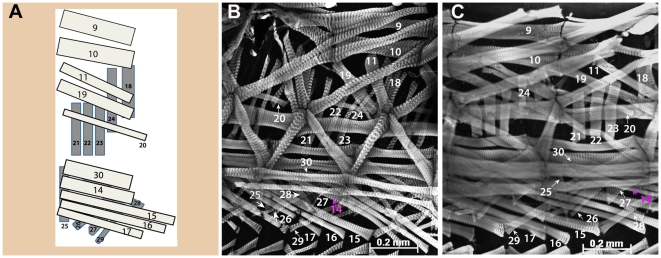
Conservation of external and intermediate body wall muscle layer in L3 larva. **A**) Scheme of external (dark gray) and intermediate (light gray) abdominal muscle layers of one hemisegment, as previously described for *D. melanogaster* (adapted from Bate, M., 1990 and 1993). **B)** *D. busckii*; **C**) *D. sechellia.* Both the external and intermediate muscle layers (grayscale) are indicated by numbers in two Drosophila species: *D. busckii* (**B**), and *D. sechellia* (**C**). Two adjacent hemisegments are shown in (**B–C**) to allow better visualization of each external and intermediate muscle fibers. Ventral, dorsal and anterior positions indicated in (**A**) also correspond to orientation of images shown in (**B**–**C**). Scale bars: 0.2 mm.

Our finding of a disproportional ratio between the mesoderm versus neuroectoderm among *Drosophilids* led us to ask how the stereotyped larval body wall musculature would be assembled and innervated by a conserved set of motoneurons. Similarly to the nerve cord development which gives rise to specific neural lineages, the *D. melanogaster* somatic myogenesis is a tightly controlled process that results in muscle fibers of unique identity. The formation of muscle fibers is initiated by the selection of a fixed number of 30 founder cells (FCs) per each hemisegment, which seeds muscle formation by fusing with surrounding myoblasts, known as Fusion-Competent Myoblasts (FCMs) [40], [41], [42]. Each founder cell has a unique identity that defines the final characteristics of the muscle fiber it will form regarding its size, orientation, innervation and attachment sites. The final muscle body wall arrangement consists of a stereotyped array of 30 muscle fibers per abdominal hemisegment (A2–A7) distributed in three separate layers: internal, intermediate and external layers (Fig. 3A and 4A) [43].

We anticipated two possible outcomes for the assembly of somatic muscle fibers of the species studied in comparison to *D. melanogaster*. The first one would be that the reduction in the number of muscle precursor cells observed in *D. busckii* and *D. pseudoobscura* would lead to the loss of muscle fibers, while an increase of muscle precursors in *D. sechellia* and *D. simulans* would lead to the formation of novel muscle fibers. If that were the case, these species would have either less or more than the 30 fibers per abdominal hemisegment observed in *D. melanogaster* [19]. Similar gain or loss of fibers in Drosophilid lineages has been previously reported for the Muscle of Lawrence, an adult male-specific muscle [44]. Alternatively, there could be a case in which the overall muscle pattern were maintained in all species, but each muscle fiber were either smaller or larger than the *D*. *melanogaster* fibers, in terms of numbers of myoblasts fused to a single fiber.

To distinguish between the two possibilities above, we analyzed the muscle body wall layers of abdominal regions encompassing segments A3 to A6 in L3 larva. Staining of muscle fibers with Phalloidin revealed that despite the increase or decrease in the number of mesodermal precursor cells, the abdominal muscle fibers of all species share an evolutionarily conserved pattern (Fig. 3 and 4). According to previously described anatomy for *D. melanogaster* [43], we identified all abdominal muscle fibers that compose the internal layer in *D*. *busckii, D. pseudoobscura*, *D. simulans* and *D. sechellia* in larval stage L3 (Fig. 3A–F). Likewise, the external and intermediate muscle layers also displayed a pattern identical to *D. melanogaster*, as shown here for the two species with the highest variation in embryo size and mesodermal domain, *D. busckii* and *D. sechellia* (Fig. 4A–C). The external and intermediate muscle layers were also analyzed in the other species and the same pattern was confirmed (data not shown). Thus, each species share the same stereotyped muscle pattern composed of fibers of equivalent identity to those of *D. melanogaster*, as judged by their identical positions, orientation, and attachment sites.

### Axon bundles display a stereotyped branching of motoneuron projections and innervations patterns into the body wall

**Figure 5:**
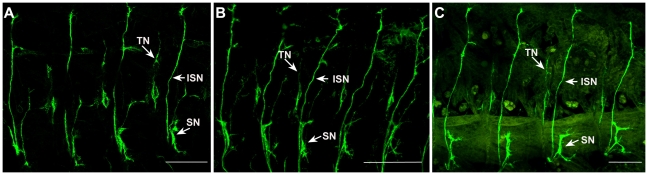
Peripheral motor nerves display stereotyped trajectories and innervation points at late embryonic stages. Anti-Fas II staining in E16–17 embryos from (**A**) *D. melanogaster*; **B**) *D. busckii*; and (**C**) *D. sechellia*. Major peripheral nerves indicated by ISN, SN, and TN have similar organization and branch at similar positions in all species analyzed. Lateral view of dissected embryo fillets, anterior to left, dorsal is up. Scale bar: 100 µm.

Another criterion used in *D. melanogaster* for identifying individual muscle fibers is to use markers to visualize the projections of main nerves that exit the ventral nerve cord and target specific muscles, forming the Neuromuscular Junctions (NMJ) [19], [45], [46], [47]. We confirmed that the stereotyped pattern of muscle fibers seen in the four species is indeed accompanied by equivalent axonal projections and targeting sites of motoneurons in both late stage E17 embryos and larva. The main axon bundles that exit the central nervous system (ISN, SN, TN) project and branch into the muscle body wall at corresponding positions in *D. melanogaster, D. busckii* and *D. sechellia* (Fig. 5A–C). At late L3 larval stages, those three primary nerves target corresponding groups of muscle fibers in all species (Fig. S2). Such remarkable similarity of innervations patterns in the species analyzed is consistent with a muscle organization akin to that of *D. melanogaster*, since mutants that eliminate or duplicate muscle fibers have abnormal motoneuron targeting [48].

### Increase in mesodermal precursors is compensated by species-specific rates of myoblast fusion

Since the pattern of the body wall musculature appears invariable across species, we tested whether the observed variation in mesodermal precursors would instead lead to the formation of larger or smaller individual muscle fibers. Here we refer to muscle size as the total number of myoblasts that have fused to a single syncytial fiber. As mentioned before, during myogenesis, founder cells recruit FCMs to fuse and form a syncytial multinucleated fiber [40], [41]. There is evidence that a given founder cell of unique identity is programmed to recruit a constant number of FCMs, even in mutant situations in which an excess of FCMs is available [49]. The number of fusion events depends on the specific muscle fiber. For instance, some of the smallest muscle fibers have as few as three to four nuclei, while the largest fibers have as many as twenty five nuclei [19]. The myoblast fusion process is completed by 13 hours after egg-laying, and after that point, the formation of muscle fibers is complete and remains unchanged until the end of larval stage without any additional fusion of myoblasts into mature fibers [19]. Therefore, the number of nuclei per fiber at larval stages reflects the number of myoblasts that were fused together in embryonic stages.

**Figure 6:**
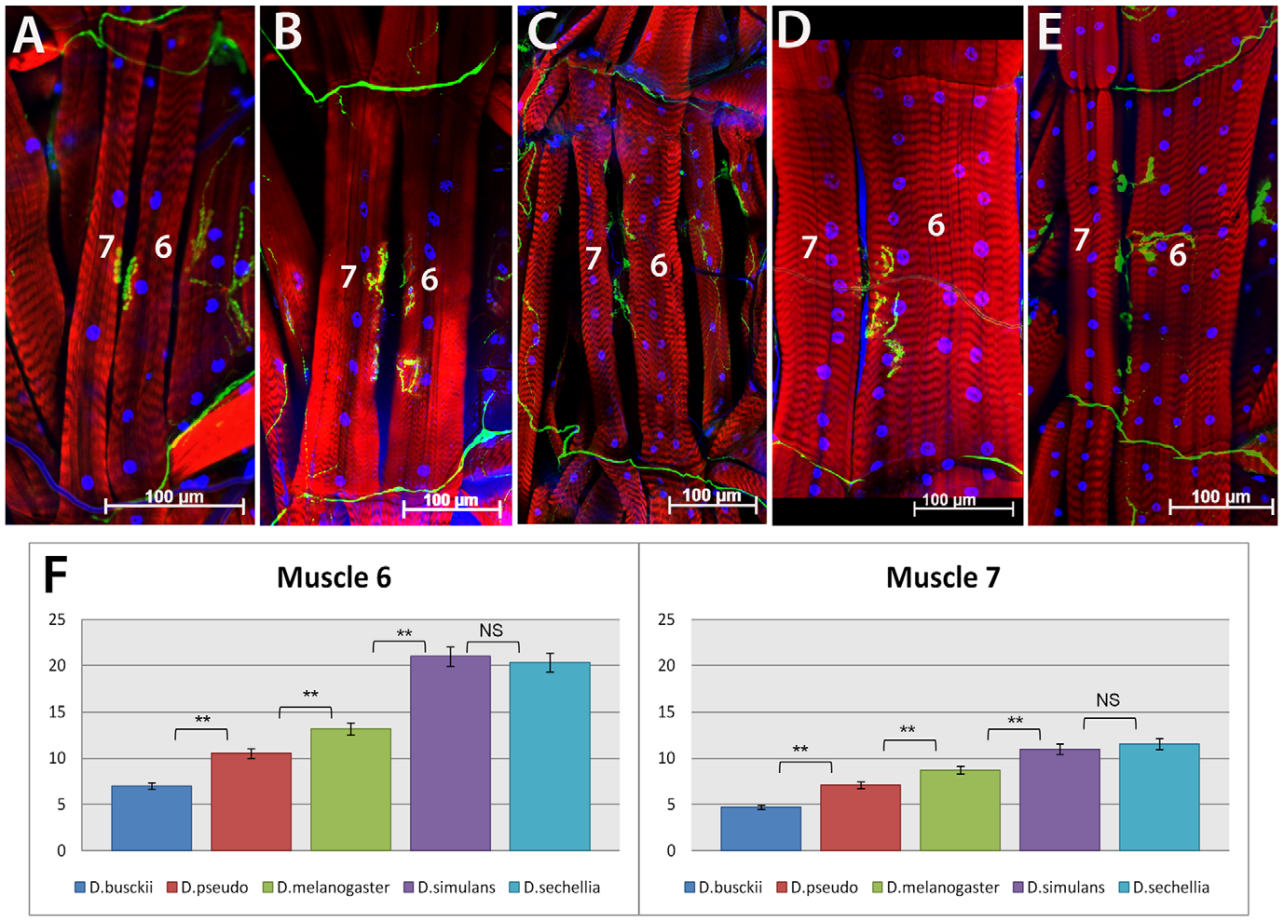
Variation in number of nuclei for muscle fibers 6 and 7 in Drosophilid larva. **A**) *D. busckii*; **B**) *D. pseudoobscura*; **C**) *D. melanogaster*; **D**) *D. simulans* and (**E**) *D. sechellia* abdominal muscle fibers 6 and 7 in L3 stage larva. The muscle fibers were stained with phalloidin (red), neuromuscular junctions with anti-HRP (green) and nuclei with Hoechst (blue). *D. busckii* and *D. pseudoobscura* (**A**, **B**) have muscle 6 and 7 reduced in size compared to *D. melanogaster* (**C**), while *D. simulans* and *D. sechellia* (**D**, **E**) have the largest fibers of all species analyzed. **F**) Quantification graph of nuclei counts per fiber 6 and 7. Species are indicated in legend. **NS**, “no statistically significant difference”. Asterisks ** indicate a *p* value<0.001 (See text for exact values). Scale bars: 100 µm. Anterior side is up.

To compare nuclei numbers per fiber among the species, we selected muscle fibers 6 and 7, which are easily identifiable and their nuclei counts were described in detail for *D. melanogaster* [50]. We focused our analysis on the abdominal segments A3 through A6, since there is little variation in muscle size among those segments, in contrast to other segments which are differentially regulated by HOX genes [51]. Our data indicate that fibers 6 and 7 of *D. busckii* (Fig. 6A) have an average of 7 nuclei (number of fibers counted n_f6_  = 7) and 4.7 nuclei (n_f7_ = 7), respectively (Fig. 6F, Table 1). The number of nuclei for *D. pseudoobscura* (Fig. 6B) has an average of 10.5 nuclei per fiber 6 (n_f6_ = 13), and 7 nuclei per fiber 7 (n_f7_ = 13). Both *D. busckii* and *D. pseudoobscura* have significantly fewer nuclei in these two fibers compared to *D. melanogaster,* for which we find an average of 13 and 8.7 nuclei per fiber, respectively (Fig. 6C, F; n_f6_ and n_f7_ = 11). In contrast, there is a significant increase in nuclei numbers in both *D. simulans* and *D. sechellia* (Fig. 6D–F; Table 1), with an average of 21 and 20.3 nuclei for fiber 6, and an average of 11 and 11.5 for fiber 7 (n_f6_ and n_f7_ = 11), respectively. The Wilcoxon rank-sum test shows that the differences observed are statistically significant, since the p-values for the comparison between *D. busckii* and *D. pseudoobscura* is 0.0003598 and for *D. pseudoobscura* and *D. melanogaster* the p-value is 0.004116. The same test confirms that the nuclei counts for *D. sechellia* has increased in comparison to *D. melanogaster* (p = 0.000140) and that there is no statistically significant difference between nuclei counts of *D. simulans* and *D. sechellia* (p = 0.4864). In addition to changes in muscle size across species, we also note that the proportion in size between muscle fibers 6 and 7 is not constant among the species analyzed. In *D. sechellia* and *D. simulans*, fiber 6 has more than twice as many nuclei than fiber 7. In contrast, in *D. busckii* and *D. pseudoobscura* such difference is not as great, and the two fibers have nearly the same number of nuclei (Fig. 6F).

**Table 1:** Muscle fiber nuclei average counts with their respective standard deviations.

Species	*D. busckii*	*D. pseudo*	*D. mel*	*D. simulans*	*D. sechellia*
Muscle fiber 6	7±1	10.53±1.89	13.18±2.52	21±1.94	20.36±3.64
Muscle fiber 7	4.71±0.48	7.07±0.95	8.52±1	11±0.89	11.54±1.69
# of segments analyzed	7	13	11	11	11

Finally, one additional muscle fiber was also included in our analysis, the intermediate muscle fiber 4, which essentially replicated the results described above for *D. busckii*, *D. melanogaster* and *D. sechellia* (Fig. S3).

## Discussion

### Dorso-ventral scaling is unequal across germ layers

The polarization of the D/V axis represents one of the most conserved features in bilaterian organisms. Despite the fact that D/V patterning has been extensively studied over the years and detailed knowledge was garnered regarding the signaling mechanisms that control gene expression and cell fate specification in both invertebrate and vertebrate models, little is known about how this system responds to changes in embryonic size. Here we investigated this issue by examining closely related species of *Drosophila* that vary in egg size. The general expectation was that changes in the distances over which the Dorsal and the Dpp/BMP gradients are established would affect all germ layers that are defined by those gradients [4], [5]. In contrast, we found that distinct germ layers respond quite differently to scaling.

Our data shows that within evolutionary distances as short as 5 mya of divergence time, there can be extreme variations in mesoderm specification. In most species analyzed, the width of mesodermal domain decreases or increases according to embryo size. However, this is not an absolute rule as *D. simulans* has a larger mesodermal domain than *D. melanogaster*, despite the fact that those two species vary less in their DV axis ([3]; Chadha and Mizutani, unpublished data).

In contrast to the plasticity seen in the mesoderm specification, the width of the neuroectoderm remains constant across species. This latter result is in agreement with two important findings regarding the development of the ventral nerve cord. First, neuroblast maps are nearly identical in a broad range of insect species [17], [18], sharing similarities even with the crustacean phylum [52]. Second, experiments of genetic manipulation that altered the width of D/V expression domains within the neuroectoderm resulted in the duplication or elimination of neuroblasts of particular identity [25], [26], [27], [28], [29], [30]. Thus, the stable width in the neuroectoderm appears to be essential for the generation of correct neural lineages and axonal scaffolds within *Drosophila* species. However, the mechanisms that protect the neuroectoderm from scaling effects remain elusive.

### Distortions in mesoderm specification can be corrected by two cellular mechanisms

Based on the stereotyped arrays of muscle fibers and innervation patterns observed in the different species, our data indicate that the mesodermal alterations can be compensated later in development. These corrections would involve an invariable selection of 30 FCs per hemisegment, and a variable rate of myoblast fusion that allows more cells to be incorporated to each muscle fiber. These two cellular mechanisms cooperate and prevent supernumerary or lack of muscle fibers.

What protects the development of the somatic body wall musculature from variations in the mesoderm size? Here we highlight some key differences between the myogenesis and neurogenesis that may explain why the assembly of the somatic body wall has more alternate ways to cope with the early variations in mesodermal specification than does the ventral nerve cord.

The initial steps of both myogenesis and neurogenesis are similar and rely on the formation of groups of equivalent cells, the promuscular and proneural groups, from which a single progenitor cell is selected through lateral inhibition [11], [12], [53], [54]. In the case of the neural progenitor cell, or neuroblast, its identity is determined once it delaminates from the proneural group, when it initiates stereotyped divisions giving rise to a defined number and types of neurons/glial cells [11], [12]. In contrast, the progenitor of somatic muscles undergoes additional asymmetric cell divisions before it gives rise to FCs and adult muscle progenitor cells. Thus, modifications in the specification of muscle progenitor cells and/or their asymmetric cell divisions could generate an identical outcome of 30 embryonic FCs in the different *Drosophila* species.

Another difference between mesodermal and neural tissue specification is the fact that the entire neuroectodermal domain contributes to the formation of a stereotyped tissue, whereas the mesodermal domain is further subdivided and gives rise to non-stereotyped tissues as well, such as the fat body, hematopoietic system and visceral musculature. Therefore, species with reduced mesodermal domain might still be able to assemble the same numbers of promuscular groups at the expense of other mesodermal precursor cells that form non-stereotyped tissues.

Finally, the present study reveals that the myoblast fusion step, which is unique to myogenesis, is an important compensatory mechanism for the formation of the somatic body wall musculature, as discussed below.

### Evolutionary changes in myoblast fusion rate compensates for mesodermal variation

Our data shows that during myogenesis of *D. busckii* and *D. pseudoobscura,* fewer myoblasts are fused together to form slender muscle fibers in comparison to *D. melanogaster*. In contrast, more myoblasts fuse into single fibers in *D. simulans* and *D. sechellia*, resulting in fibers of increased size. The differential regulation of fusion events appears to be the only characteristic of FC identity that is unique to each species.

One of the main regulators of myoblast fusion is the adhesion molecule Kin of Irre/Dumbfounded (Kirre/Duf), which is expressed exclusively by FCs and functions as an attractant to FCMs [55], [56]. The expression of *kirre/duf* is down regulated once the correct number of fused FCMs is achieved for a given muscle fiber [55]. If this down regulation of *kirre/duf* is modulated by the number of FCM that are aggregated, then there are two ways of increasing myoblast fusion. One would be if inhibitory signals released from fused FCMs are weaker in strength and the other would be if the sensitivity of *kirre/duf* to these signals is lower. In either case, more myoblasts would be added to the fiber. Recently, the *cis-*regulatory region of *kirre/duf* gene was identified in a group of *Drosophila* species, including *D. pseudoobscura* and *D. simulans,* and was found to have stretches of sequence divergence [57]. These results support the view that modifications in the cis-regulatory sequence of *kirre/duf* could be responsible for different rates of myoblast fusion observed in these *Drosophila* species. However, further tests would be needed to determine whether constructs with *kirre/duf* from *D. simulans* and *D. pseudoobscura* inserted in *D. melanogaster* respond as expected by creating fibers with more or less myoblasts, respectively.

### Evolution of embryo size and correction mechanisms involved in fast evolution of genes involved in DV patterning and myogenesis

Variations in embryo size impose challenges to developing organisms, which must be overcome to ensure viability. In all species investigated in this study, some separated by several million years and others by only several thousand years, we note that alterations in mesodermal size were resolved by a common mechanism that increases or decreases the rate of myoblast fusion to generate the same stereotyped array of muscles. Since the variation in mesodermal domain and myoblast fusion rates occured within very short evolutionary distances, these are fast evolving traits. Consistent with this view, there is evidence from the literature that genes belonging to the Toll and Dorsal/NFkB pathway, which participate in both immune response and D/V patterning, are fast evolving within twelve Drosophila species [59], [60]. This finding can be explained as adaptation to new pathogens found in the particular niches these species occupy. However, a recent comparison of the genomes of three *melanogaster* sister species identified components of the Dorsal/NFkB pathway that diverged the most in *D. melanogaster,* but the least in the pair *D. simulans/D. sechellia*, despite the fact that the latter two species do occupy completely different niches (i.e. one is cosmopolitan and the other is restricted to the plant Morinda, respectively) [58]. These data provide further evidence that D/V patterning itself, and not only immunity, evolves fast and point out to specific candidates in the Dorsal/NFkB pathway undergoing those changes.

We recently surveyed the genes conserved in the *D. simulans/D. sechellia* pair that diverged from *D. melanogaster* and identified genes exclusively expressed within either the mesoderm or somatic muscle fibers that are currently being investigated (CMM et al. in preparation). Thus, together these data suggests that it is possible that *in silico* comparisons across these species will reveal additional components of the myogenesis regulatory network [61], [62], [63], [64], which may also function to correct distortions generated by embryonic size variation.

## Materials and Methods

### Drosophila strains

The following strains were used*: D. melanogaster* (Oregon R, wild type) *D. sechellia* (*Zn^1^; v^1^; f^1^,* Species Center at UCSD, stock number 14021-04248-19), *D. pseudoobscura* (wild type, K-S12, KYORIN Stock Center), *D. busckii* (Species Center at UCSD, stock number 300-0081-23) and *D. simulans* (wild type, Species Center at UCSD, stock number 14021-0251.199). Flies were reared at 25^°^C in standard corn meal and molasses media. For *D. busckii*, the media was supplemented with a thick layer of yeast paste and for *D. sechellia*, a supplement with Noni leather (dry Morinda fruit) was used.

### Tissue preparation, immunostaining and microscopy

Third instar larvae were dissected according to protocol described previously [65]. Larval tissue was then fixed in 4% formaldehyde for 20 minutes, rinsed in PBT several times, and blocked in 5% to 10% WBS (Western Blocking Solution, Roche) in PBT for 30 minutes. Next, the fixed tissue was incubated with primary antibodies diluted in 5%–10% WBS/PBT for 30 to 40 minutes at RT, washed thoroughly for 30 minutes and then incubated with secondary antibodies for 30 to 45 min at RT. Embryos were collected, fixed and processed for *in situ hybridization* and immunohistochemistry according to [66]. The following reagents were used: Goat anti-Horseradish Peroxidase (anti-HRP, ICN Biomedical) at the concentration of 1∶500 for staining axonal projections and terminal boutons; Phalloidin conjugated with Rhodamine at 1∶100 (Cytoskeleton) for staining actin filaments; Donkey anti-Goat Alexa 488 at 1∶500 (Molecular Probes, Invitrogen); Rabbit anti-DMEF2 at the concentration of 1∶ 1500 (kindly provided by Dr. Hanh T. Nguyen) [37]; Donkey anti-Rabbit Alexa 555 at 1∶500 (Molecular Probes, Invitrogen). RNA labeled probes used were *sna*-Digoxigenin and *sog*-Biotin, detected with Mouse anti-Biotin and Sheep anti-Digoxigenin (Roche) at 1∶1000, and secondary antibodies Donkey anti-Mouse Alexa 647 and Donkey anti- Sheep Alexa 488 (Molecular Probes, Invitrogen) at the concentration of 1∶500. For nuclei staining, Hoechst was used at the concentration of 10 µg/ml (Molecular Probes, Invitrogen). Cross-sections of embryos were made by hand within the trunk region [67], using a micro-dissecting knife (Roboz). Embryos and dissected larvae were mounted in Slow Fade or Prolong mounting media (Molecular Probes, Invitrogen) and images were collected on a LSM700 Zeiss Confocal Microscope.

### Statistical analysis

The nuclei of muscle fibers 4, 6 and 7 within abdominal segments A3 through A5 were counted using the counting tool of Adobe Photoshop CS3 program. The data obtained was statistically analyzed by using Wilcoxonh rank-sum test. The sample sizes and p-values obtained are indicated in text.

## Supporting Information

Figure S1 **Evolutionary change in mesoderm size.** Cross-section of embryos from five different *Drosophila* species stained for *sog* (red), *sna* (green) and Hoescht nuclear dye (blue). From left to right, *D. busckii, D. pseudoobscura, D. melanogaster, D. simulans* and *D. sechellia*. Scale bar: 100 µm.
(TIF)

Figure S2 **Innnervation patterns of larval abdominal muscle fibers in Drosophilids. A**) Schematic drawing according to [48] depicting a single abdominal hemisegment and the primary three nerves that project into the body wall musculature, as described for D. *melanogaster*. The motoneurons are color coded in red (**TN**, transverse nerve), blue (**ISN**, intersegmental nerve) and orange (**SN**, segmental nerve). Tissue preparations of muscle body wall showing two abdominal hemisegments of *D. melanogaster* (**B**), *D. busckii* (**C**), *D. pseudoobscura* (**D**); *D. simulans* (**E**) and *D. sechellia* (**F**) showing the same motoneurons TN, ISN, SN (arrows) and their internal muscle targets (muscle fibers 6, 8, 4 and 2 are indicated in **B–F**). Muscle fibers were stained with Phalloidin (red) and the motoneurons with anti-HRP antibody (green). Ventral, dorsal and anterior positions are indicated in (**A**) and also correspond to orientation of images shown in (**B–F**). Scale bar: 100 µm.
(TIF)

Figure S3 **Differences in number of fused myoblasts in larval muscle fiber 4 across Drosophila species.** Larval tissue preparation of *D busckii* (**A**), *D. melanogaster* (**B**) and *D. sechellia* (**C**). The muscle fibers were stained with Phalloidin (red) and the nuclei with Hoechst (blue). (D) Graph of nuclei counts per muscle fiber 4 in the three species above.
(TIF)
