# Supplementary figures and images for: Variation in Mesoderm Specification across Drosophilids Is Compensated by Different Rates of Myoblast Fusion during Body Wall Musculature Development
Source: PLoS One. 2011 Dec 14;6(12):e28970. doi: 10.1371/journal.pone.0028970 (PMC3237579; doi:10.1371/journal.pone.0028970)

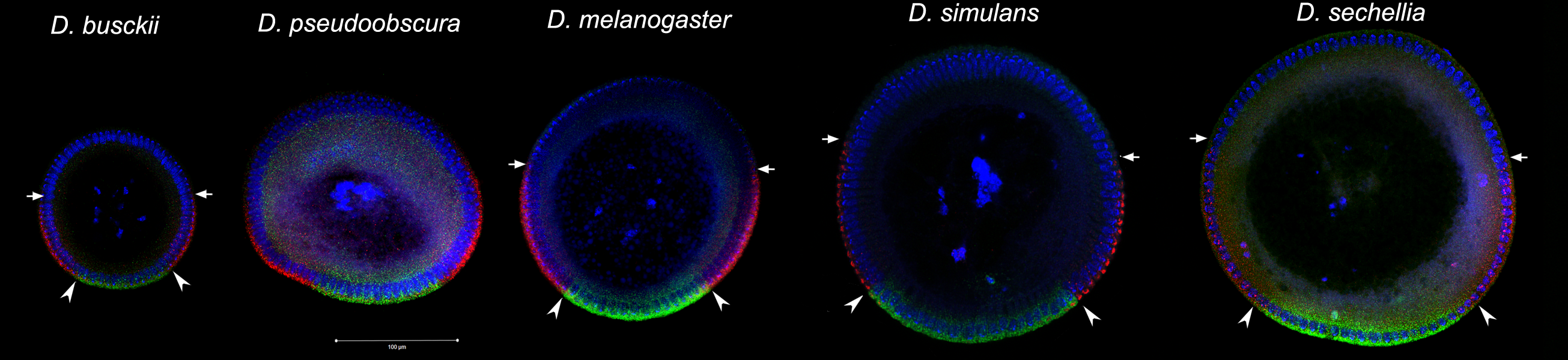

Supplement: Figure S1 — Evolutionary change in mesoderm size. Cross-section of embryos from five different Drosophila species stained for sog (red), sna (green) and Hoescht nuclear dye (blue). From left to right, D. busckii, D. pseudoobscura, D. melanogaster, D. simulans and D. sechellia. Scale bar: 100 µm. (TIF) [file pone.0028970.s001.tif]

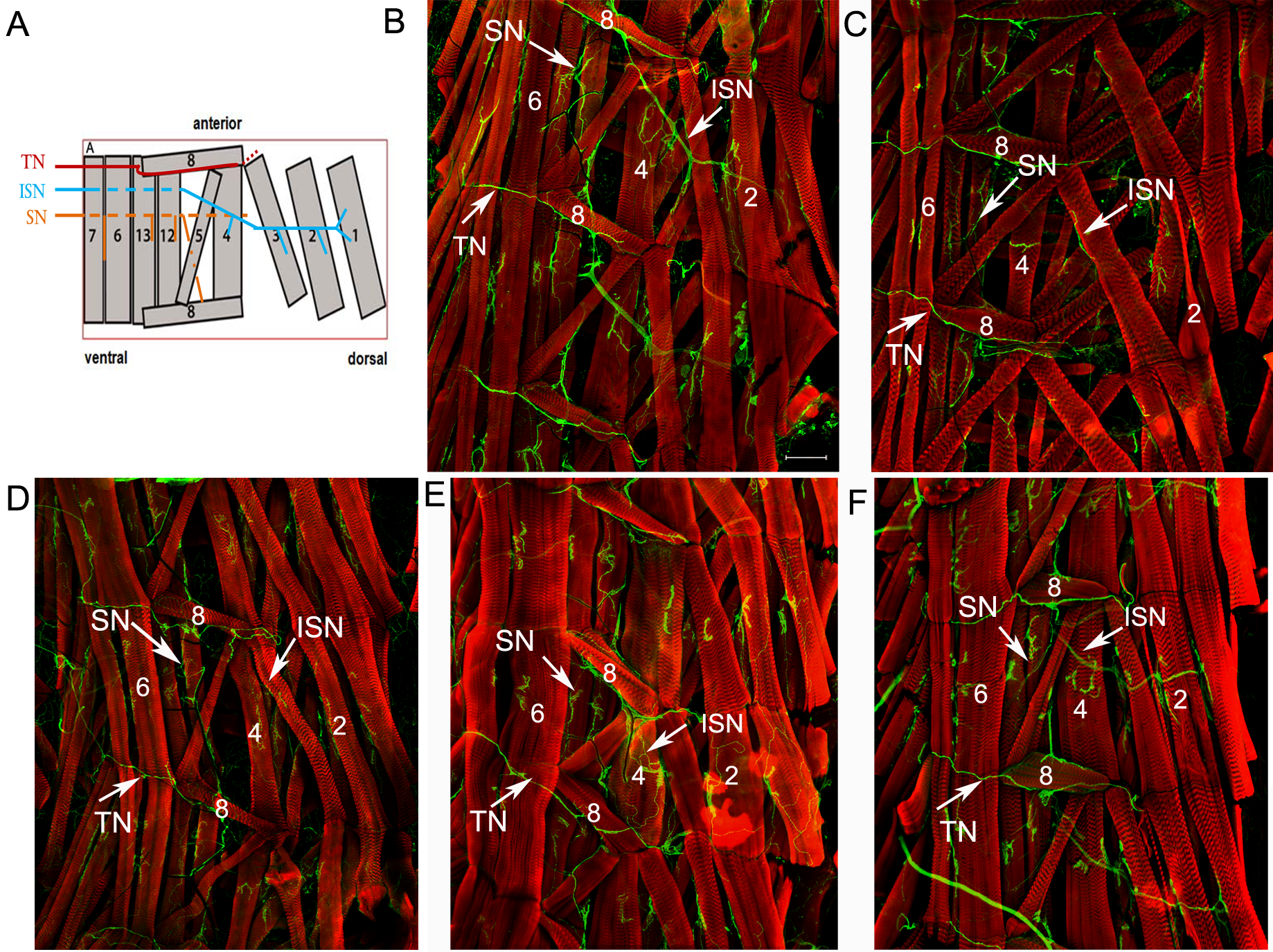

Supplement: Figure S2 — Innnervation patterns of larval abdominal muscle fibers in Drosophilids. A) Schematic drawing according to [48] depicting a single abdominal hemisegment and the primary three nerves that project into the body wall musculature, as described for D. melanogaster. The motoneurons are color coded in red (TN, transverse nerve), blue (ISN, intersegmental nerve) and orange (SN, segmental nerve). Tissue preparations of muscle body wall showing two abdominal hemisegments of D. melanogaster (B), D. busckii (C), D. pseudoobscura (D); D. simulans (E) and D. sechellia (F) showing the same motoneurons TN, ISN, SN (arrows) and their internal muscle targets (muscle fibers 6, 8, 4 and 2 are indicated in B–F). Muscle fibers were stained with Phalloidin (red) and the motoneurons with anti-HRP antibody (green). Ventral, dorsal and anterior positions are indicated in (A) and also correspond to orientation of images shown in (B–F). Scale bar: 100 µm. (TIF) [file pone.0028970.s002.tif]

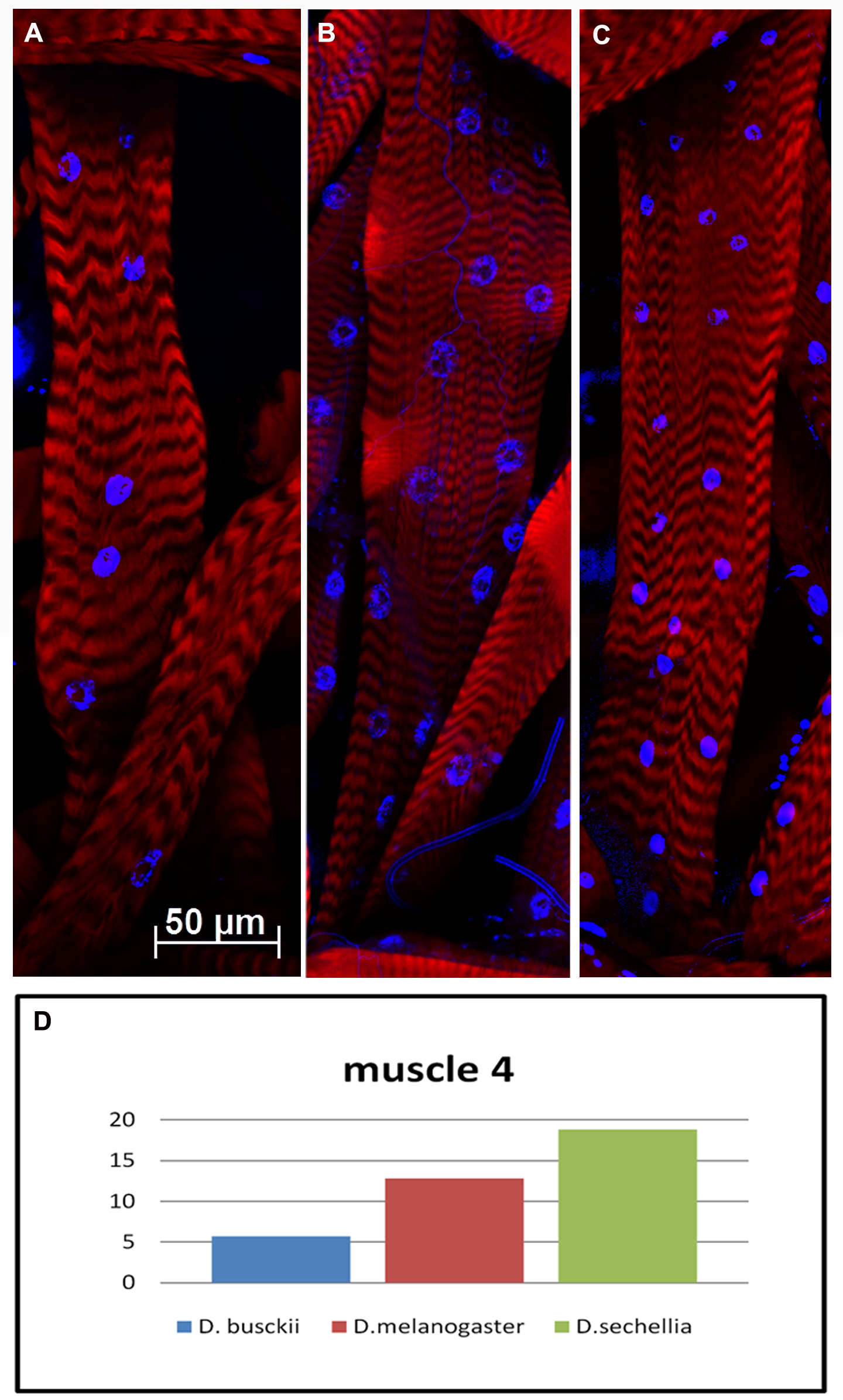

Supplement: Figure S3 — Differences in number of fused myoblasts in larval muscle fiber 4 across Drosophila species. Larval tissue preparation of D busckii (A), D. melanogaster (B) and D. sechellia (C). The muscle fibers were stained with Phalloidin (red) and the nuclei with Hoechst (blue). (D) Graph of nuclei counts per muscle fiber 4 in the three species above. (TIF) [file pone.0028970.s003.tif]
